# Magnetic field control of charge excitations in CoFe_2_O_4_

**DOI:** 10.1063/1.5021792

**Published:** 2018-06-01

**Authors:** Brian S. Holinsworth, Nathan C. Harms, Shiyu Fan, Dipanjan Mazumdar, Arun Gupta, Stephen A. McGill, Janice L. Musfeldt

**Affiliations:** 1 Department of Chemistry, University of Tennessee, Knoxville, Tennessee 37996, USA; 2 Department of Physics, University of Tennessee, Knoxville, Tennessee 37996, USA; 3 Center for Materials for Information Technology, University of Alabama, Tuscaloosa, Alabama 35487, USA; 4 National High Magnetic Field Laboratory, Florida State University, Tallahassee, Florida 32310, USA

## Abstract

We combine magnetic circular dichroism and photoconductivity with prior optical absorption and first principles calculations to unravel spin-charge interactions in the high Curie temperature magnet CoFe_2_O_4_. In addition to revising the bandgap hierarchy, we reveal a broad set of charge transfer excitations in the spin down channel which are sensitive to the metamagnetic transition involving the spin state on Co centers. We also show photoconductivity that depends on an applied magnetic field. These findings open the door for the creation and control of spin-polarized electronic excitations from the minority channel charge transfer in spinel ferrites and other earth-abundant materials.

Multifunctional, high Curie temperature magnetic semiconductors are tailor-made for modern device applications. They naturally provide sizable magnetic moments, switchable spin states, and spin-selective bandgaps for use in spintronics, spin-caloritronics, and straintronics.^1–4^ Moreover, the use of the spin rather than the charge is crucial for the development of ultra-low power devices because there is less heat to dissipate. Among the various candidate materials, iron oxides are well studied, sustainable, and earth-abundant. The spinel ferrites, with general formula *A*Fe_2_O_4_, are particularly attractive with CoFe_2_O_4_ and NiFe_2_O_4_ as flagship examples.

CoFe_2_O_4_ is well-known as a magnetic semiconductor with a typical *A*B_2_O_4_ spinel crystal structure (space group $Fd\bar{3}m$, No.: 227) (Fig. 1).^5^ This system has an inversion fraction *λ* of ≈0.75, so an explicit rendering can be written as {Co_0.25_Fe_0.75_}_tet_[Co_0.75_Fe_1.25_]_oct_O_4_.^6,7^ Here, {}_tet_ refers to the tetrahedral site and []_oct_ refers to the octahedral site.^8^ By comparison, NiFe_2_O_4_ is a fully inverse spinel.^9,10^ The Curie temperature, *T*_C_, is 795 K,^11^ and the coercivity and saturation magnetization are 1.1 T and 450 emu/cm^3^, respectively.^12^ The saturation of the Co moments occurs at *B*_*s*,Co_ ≈ 3 T.^12^ Thus, an applied field drives the system from a ↓↓↑ to ↑↓↑ configuration and vice versa, upon field reversal [Fig. 1(d)]. This sequence refers to spins on the Co site, the Fe octahedral site, and the Fe tetrahedral site, respectively. The field therefore selects one magnetic state over another as the Co spin flips. Presumably, the iron moments saturate at even higher magnetic fields (giving the ↑↑↑ configuration), although the exact value of *B*_*s*,Fe_ has not yet been measured. Confinement and strain provide additional control of the magnetic state.^13^ The magnetocrystalline anisotropy of CoFe_2_O_4_ is 2 × 10^6^ ergs/cm^3^,^14^ and the magnetostrictive coefficient along the [100] direction is large: −5.90 × 10^−4^.^15–17^ Together, these properties have led to contemporary usage in spin-filtering heterostructures, composite multiferroics, and embedded nano-structures.^12,18–22^

**FIG. 1.**
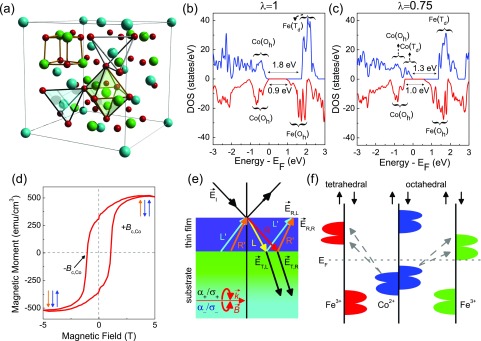
(a) Crystal structure of CoFe_2_O_4_ (space group $Fd\bar{3}m$, No.: 227).^5^ [(b) and (c)] Calculated electronic structures of CoFe_2_O_4_ from Ref. 23 carried out using *LSDA* + *U* with U_*eff*_ = 4.5 eV for Fe and 4.0 eV for Co for the fully inverse and *λ* = 0.75 cases, respectively. The bandgaps in the minority and majority channel are labeled. (d) Magnetization data from Ref. 12 show the hysteresis loop, the 1.1 T coercive field, and how the ↓↓↑ and ↑↓↑ states are switched across *B*_*c*,Co_. (e) Schematic of our magneto-optical experiments. The Faraday measurement geometry is on the bottom left of the panel. In general, the wave vectors for right- and left-circularly polarized light will differ. (f) Schematic view of the density of states. The excitations fall into two categories: (i) intersublattice charge transfer (Co(*O*_*h*_) → Fe(*T*_*d*_)) and (ii) intervalence charge transfer (Co(*O*_*h*_) → Fe(*O*_*h*_)). The color scheme denotes different sublattices.

Recent work uncovers fascinating electronic properties as well.^11,15,23,25^ The analysis of the spectral functions and partial densities of states [Figs. 1(b) and 1(c)] reveals sizable exchange splittings, a fundamental indirect bandgap, and the possibility of spin-polarized current emanating from low energy minority channel excitations.^23^ Importantly, CoFe_2_O_4_ has a lower electronic energy scale compared to similar materials like NiFe_2_O_4_ and Co:ZnO.^23,26,27^ Our recent spectroscopic work on epitaxial thin films of CoFe_2_O_4_ uncovers a 1.2 eV indirect gap, a hierarchy of higher energy direct gaps, and a favorable overlap with the solar spectrum.^23^ These findings raise questions about broader aspects of the electronic structure in CoFe_2_O_4_ and the Ni analog, for instance, what are the band polarizations that contribute to magnetism, and how does the *I*–*V* curve respond to light? These issues are central to advancing the microscopic understanding of high *T*_C_ magnetic oxides and their many applications.

Spinel ferrites are also well-suited to the development of structure-property relations.^28–30^ Just as in perovskites, transition metal centers bring in the electron correlation, anisotropy, and control charge, spin, and local lattice environment. To the first order, the charge, spin, orbital, and lattice channels operate independently, although their entanglement leads to compelling interactions along with opportunities for property control under external stimuli.^31–34^ At the same time, spinel ferrites sport degrees of freedom that reach beyond those in perovskites, e.g., the cation inversion parameter *λ*.^35–37^ This provides a framework for the development of new and useful properties as well as novel physics.

In this work, we bring together magnetic circular dichroism (MCD) and photoconductivity to investigate entangled electronic and magnetic degrees of freedom in the spinel ferrite CoFe_2_O_4_. Our objective is to determine the spin polarization and the rotation (which is proportional to magnetization) and by so doing uncover the bands and charges that are responsible for the unique magnetic properties. Even though there has been other magneto-spectroscopy of spinels,^38,39^ to our knowledge, there has been no work on these issues—an important oversight considering the very real application potential of these compounds. Analysis reveals (i) a broad energy window of purely minority channel excitations that overlaps well with the solar spectrum, (ii) magnetic field tunability of these states that derives from field-induced switching of the spin state and the spin-charge coupling in this system, and (iii) enhanced photoconductivity under the applied magnetic field. Comparison with the Ni analog^23,40^ also allows the development of several important structure-property relations particularly with regard to the role of the inversion fraction. Taken together, we uncover an energy window in the electronic structure where light generates spin-polarized carriers and where the magnetic field influences the relevant charge excitations. We discuss how high temperature magnets like CoFe_2_O_4_ and NiFe_2_O_4_ may offer new opportunities for light harvesting and oxide electronics.^41,42^

High-quality epitaxial CoFe_2_O_4_ films (30–200 nm) were grown on (001)-orientated MgAl_2_O_4_ substrates via pulsed laser deposition as described previously.^12^ The different thicknesses allowed for the control of optical density. MCD measurements were carried out at the National High Magnetic Field Laboratory using a 300 W Xe lamp, an 0.25 m monochromator, a purpose-built transmittance probe, and a 10 T superconducting magnet. Importantly, the MCD measurements were done in the Faraday geometry, that is, $\vec{k}$ and $\vec{B}$ are parallel/anti-parallel, depending upon the sign of $\vec{B}$. The signal-to-noise ratio was increased by chopping the light; likewise, by passing linearly polarized light through a photoelastic modulator, right-circularly polarized light and left-circularly polarized light were dynamically separated as *δ*(*t*) = *λ*/4 sin(*ωt*).^43^ All signals were separated by lock-in amplifiers. In addition to the epitaxial CoFe_2_O_4_ films, we also measured the dichroic response of MgAl_2_O_4_ as a function of energy and magnetic field. No magnetic field dependence of the substrate was observed at any energy (see the supplementary material). Photoconductivity measurements were carried out on a home-built setup equipped with a Xe lamp, a series of narrow bandpass filters, a high voltage source, and a 1.5 T magnet. Sputtered gold contacts along with silver epoxy and 75 *μ*m wires were used as contacts. The photoconductance was normalized by power density at each measurement wavelength and then converted to energy for comparison with the spectral data.

Figure 2(a) displays the MCD spectrum of CoFe_2_O_4_ in applied fields up to ±10 T at 1.6 K. The trends are overall systematic with increasing and decreasing fields, as expected. For comparison, we include the linear absorption spectrum (*α*(*E*)), with the 1.2 and 2.7 eV bandgaps indicated on the energy axis.^23^ Examination of the spectra in Fig. 2(a) immediately reveals a large number of states below the majority channel direct gap (2.7 eV). Moreover, local maxima in the dichroic response coincide with inflection points in the absorption spectra. This demonstrates an important derivative relationship between *I*_*MCD*_ and *α*(*E*). The magnitude of the dichroic response is often expressed as^44^

$$I_{MCD}\approx \frac{\left(\alpha_{+}\left(E\right)-\alpha_{-}\left(E\right)\right)d}{2}\approx \frac{\Delta E}{2}\frac{1}{\alpha \left(E\right)}\frac{d\alpha \left(E\right)}{dE}.$$ (1)

Here, *α*_+_(*E*) − *α*_−_(*E*) is the absorption difference between right- and left-circularly polarized light, *dα*(*E*)/*dE* is the energy derivative of absorption, Δ*E* is the change in energy of the peak position, and *d* is the film thickness. Further, the resulting contrast in *α*_±_(*E*) correlates with *σ*_±_, the helicity.^44^ Note that there is a direct proportionality between *I*_*MCD*_ and *dα*(*E*)/*dE*. Absorption is a joint density of states effect, so *I*_*MCD*_ highlights critical points in the band structure.

**FIG. 2.**
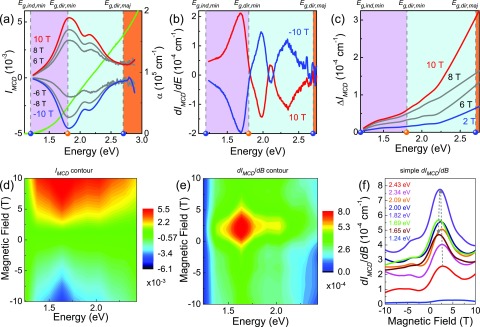
(a) MCD spectra of CoFe_2_O_4_ at 1.6 K and ±10 T along with the linear absorption (green line) for comparison. The points on the energy axis define the bandgaps, with their corresponding assignment at the top.^23^ (b) Derivative of *I*_*MCD*_ with respect to energy, emphasizing the inflection points. (c) Residual MCD signal obtained from the difference of *I*_*MCD*_ in the positive and negative field directions, Δ*I*_*MCD*_ = *I*_*MCD*_(*E*, *B*) − *I*_*MCD*_(*E*, −*B*). This corresponds to the difference between the ↑↓↑ and ↓↓↑ states. (d) Contour plot of the MCD spectrum (*I*_*MCD*_) in the energy-magnetic field plane. The data in panel (a) are a set of constant field cuts of this surface. (e) Contour plot of *dI*_*MCD*_/*dB* as a function of energy and magnetic field. (f) Constant energy cuts of *dI*_*MCD*_/*dB* vs. magnetic field plot. The data in (d)–(f) were taken on the up-sweep (−10 T → 10 T).

The direct assignment of the spectral features of CoFe_2_O_4_ comes from an understanding of the band structure and projected density of states [Figs. 1(b) and 1(c)].^23,24^ Minority-channel transitions involving hybridized Co(*O*_*h*_) + O → Fe(*O*_*h*_) are responsible for the absorption edge and the fundamental indirect gap.^23^ These transitions can be considered to be inter-sublattice charge transfer as indicated by the schematic in Fig. 1(f). The same set of transitions also gives a direct gap excitation in the spin-down channel.^23^ We confirm that it is direct from the magnitude of the absorption (1 × 10^5^ cm^−1^). The majority-channel direct gap arises from Co(*O*_*h*_) + O → Fe(*T*_*d*_) excitations. Of course, when *λ* = 0.75, this becomes Co(*O*_*h*_) + Co(*T*_*d*_) + O → Fe(*T*_*d*_).

Returning to Fig. 2(a), there are several features in the 1.5 to 2.5 eV energy window—where only minority channel charge transfer excitations are expected—indicating that there are excitations that exist solely in the spin-down channel. The lowest energy excitation, centered at 1.8 eV, presents considerable asymmetry on the low energy tail, suggesting that the nearby indirect gap excitation may be affecting the line shape. By comparison, peak fitting reveals that the excitation centered at ≈2.2 eV has the expected Lorentzian line shape (see the supplementary material). Beyond the exquisite sensitivity for locating important features in the density of states, dispersion in MCD spectra gives reliable estimates of the spin splitting between majority and minority bands. We find exchange splittings of 0.15 eV, in reasonable agreement with theoretical predictions.^23,25^

Figure 2(b) displays the derivative of the MCD spectrum *dI*_*MCD*_/*dE* as a function of energy at ±10 T. There are several intriguing features that give rise to zero-crossings near 1.2, 1.8, 2.15, and 2.7 eV. As a reminder, the indirect gap in the minority channel is at 1.2 eV, and the direct gap in the majority channel is at 2.7 eV. The energy scale at ≈1.8 eV—indicated by the node in *dI*_*MCD*_/*dE*—is also important, although it was overlooked in our prior analysis of the absorption spectrum because it was less than clear. We assign this feature as a Co (*e*_*g*_) → Fe (*t*_2*g*_) excitation in the minority channel—probably between two octahedral sites. By comparison, the zero crossing in *dI*_*MCD*_/*dE* near 2.15 eV seems to be a density of states effect. This supposition is based upon the shape of the projected density of states in this energy window. The full bandgap hierarchy in CoFe_2_O_4_ is thus 1.2 eV (indirect, minority channel), 1.8 eV (direct, minority channel), and 2.7 eV (direct, majority channel).

The MCD spectrum of CoFe_2_O_4_ is similar in magnitude to that of NiFe_2_O_4_,^40^ although in the Ni analog, the oscillator strength and the series of bandgaps are pushed to higher energies. The excitations in CoFe_2_O_4_ thus have a much better overlap with the solar spectrum from both a bandgap and density of state perspective. The fact that *λ* ≈ 0.75 in CoFe_2_O_4_ is not readily apparent from the MCD data, although as discussed earlier, it does affect the assignments. For instance, the majority-channel direct gap arises from Co(*O*_*h*_) + O → Fe(*T*_*d*_) excitations, and when *λ* = 0.75, the assignment should be considered as Co(*O*_*h*_) + Co(*T*_*d*_) + O → Fe(*T*_*d*_). The complexity of the charge transfer excitations below 2.5 eV may be responsible for the additional oscillator strength.

From the preceding discussion and Eq. (1), we see that the electronic aspects of the dichroic response of CoFe_2_O_4_ are fairly straightforward. But what about the magnetic response and what effect will a change in spin state have on *I*_*MCD*_? In other words, we know that the applied field flips the spin on the Co sites and drives a ↓↓↑ to ↑↓↑ transition at *B*_*c*,Co_ [Fig. 1(b)]. We do not, however, yet know the electronic signatures of this entanglement.

The connection between the magnetic circular dichroism and the spin state can be understood in a straightforward manner by recalling that time reversal symmetry is broken in magnetic materials. This means that separate wave vectors ${\vec{k}}_{+}$ and ${\vec{k}}_{-}$ must be used to define the propagation of right- and left-circularly polarized light [Fig. 1(e)]. This results in the development of off-diagonal elements in the complex dielectric tensor $\overset{↔}{\varepsilon }\left(E\right)$.^45,46^ In addition to separate wave vectors being required to describe the propagation of right- and left-circularly polarized light, all of the optical constants are energy dependent and tensorial in nature. For example, the complex refractive index is $\overset{↔}{n}\left(E\right)={\overset{↔}{n}}^{\prime }\left(E\right)+{\overset{↔}{n}}^{\prime \prime }\left(E\right)=\sqrt{\overset{↔}{\varepsilon }\left(E\right)\overset{↔}{\mu }\left(E\right)}$. Moreover, the extinction coefficient ${\overset{↔}{n}}^{\prime \prime }$(*E*) is proportional to absorption $\overset{↔}{\alpha }$(*E*). Therefore, off-diagonal components of the dielectric tensor (or the fact that the magnetic permeability of a magnetic material $\overset{↔}{\mu }$ is not 1.0) are directly connected to the absorption (and in turn the absorption difference between right- and left-circularly polarized light). More precisely, the information derived from the dielectric tensor, and hence the refractive indices (${\overset{↔}{n}}_{\pm }={\overset{↔}{n}}_{\pm }^{\prime }+i{\overset{↔}{n}}_{\pm }^{\prime \prime }$) for right- and left-circularly polarized light, is expressed in the relationships in Eqs. (2)–(5).^47^ Taking the *z* direction as being parallel to the magnetization $\vec{m}$, the dielectric tensor appears as the following:

$$\overset{↔}{\varepsilon }=\;\left|\begin{array}{ccc} \varepsilon_{xx} & i\varepsilon_{xy} & 0\\ -i\varepsilon_{xy} & \varepsilon_{yy} & 0\\ 0 & 0 & \varepsilon_{zz} \end{array}\right|,$$ (2)

$$\approx \;{\overset{↔}{n}}^{2}\left|\begin{array}{ccc} 1 & iQ{\vec{m}}_{z} & 0\\ -iQ{\vec{m}}_{z} & 1 & 0\\ 0 & 0 & 1 \end{array}\right|,$$ (2a)

$${\overset{↔}{\varepsilon }}_{\pm }=\;{\overset{↔}{\varepsilon }}_{xx}\pm {\overset{↔}{\varepsilon }}_{xy},$$ (3)

$${\overset{↔}{n}}_{\pm }=\;\sqrt{{\overset{↔}{\varepsilon }}_{\pm }}\approx {\overset{↔}{n}}_{0}\pm \frac{{\overset{↔}{\varepsilon }}_{xy}}{2{\overset{↔}{n}}_{0}},$$ (4)

$${\overset{↔}{n}}_{0}=\;\sqrt{{\overset{↔}{\varepsilon }}_{\pm }{\overset{↔}{\mu }}_{\pm }}.$$ (5)

These relationships demonstrate the attenuation of circularly polarized light across a medium. Here, ${\overset{↔}{n}}_{\pm }={\left(\varepsilon_{xx}\pm \varepsilon_{xy}\right)}^{1/2}$ is the refractive index, as expressed by Eqs. (2)–(5), for right- and left-circularly polarized light arising from the dielectric function $\overset{↔}{\varepsilon }$. It is also customary to define *Q* as a material-specific magneto-optic constant. The correlation between the imaginary component of the refractive index ${\overset{↔}{n}}_{\pm }^{\prime \prime }$ and absorption provides a direct correspondence between the magnetic polarization underlying the transition and the dichroic response. Therefore, an assignment of the magnetic nature of the electronic structure underpinning specific spectroscopic transitions follows logically. An important caveat to these relationships is that the nature of the excitation precludes $\overset{↔}{\mu }=1$ and thus the refractive index includes this salient component as shown in Eq. (5). This makes magnetic circular dichroism a sensitive tool for probing both electronic and magnetic properties.

Figure 2(c) shows the residual MCD signal. This quantity is defined as the difference in the MCD spectra taken in the positive and negative field directions, Δ*I*_*MCD*_ = *I*_*MCD*_(*E*, *B*) − *I*_*MCD*_(*E*, −*B*). Thus, the residual signal is differentiated from the primary signal by simple subtraction. Physically, Δ*I*_*MCD*_ represents the difference in the dichroic response between the ↓↓↑ and ↑↓↑ states. In other words, the field selects the magnetic state, and Δ*I*_*MCD*_ represents the asymmetry in the number of spin-dependent states present in the excitation upon reversing the applied magnetic field. In NiFe_2_O_4_, electronic structure calculations reveal that the Ni states reside in either the minority or majority channel depending on whether spins are in the ↓↓↑ or ↑↓↑ state.^40^ A similar swap of the Co density of states is anticipated here as the magnetic field is swept across *B*_*c*,Co_. Just as *I*_*MCD*_ quantifies the number of states involved in Co → hybridized Fe(*O*_*h*_) + Co excitations (with Co charge accessing a different set of states above the Fermi level depending on the field direction), Δ*I*_*MCD*_ reveals the small fraction of excitations that are spin independent and insensitive to field reversal. They probably involve ions other than Co, e.g., Fe and O. The overall size of the residual signal represented by Δ*I*_*MCD*_ is small. It is on the order of 10^−5^ near *B*_*c*,Co_, increasing to 10^−4^ at a full field. Overall, the MCD spectrum of CoFe_2_O_4_ is controlled by the underlying spin state (↓↓↑ or ↑↓↑) and spin-charge interactions. The use of a small (rather than large) field to flip the Co spins obviously assures a large, controllable primary signal and a modest residual signal.

To further explore the energy and magnetic field dependence of the dichroic response of CoFe_2_O_4_, we created contour plots of these spectra. The data in Fig. 2(a) are thus a set of constant field cuts through the contour plot of Fig. 2(d). Examination of *I*_*MCD*_ in the contour format reveals that the slope reaches a maximum near 2.5 or 3 T depending upon the energy. This suggests that a more detailed analysis of this edge may provide useful information about how the electronic excitations depend upon the spin state (and how they change across the coercive field). Figure 2(e) displays *dI*_*MCD*_/*dB*, as a function of energy and magnetic field. The largest changes are between 1.5 and 2.1 eV. This indicates that low energy charge transfer excitations are most strongly correlated with the spin state as well as with spin-polarized absorption. Figure 2(f) cuts the *dI*_*MCD*_/*dB* data in the contour plot at selected energies. Again, we see that changes are most pronounced between 1.5 and 2.1 eV (where the mixed state transitions in the minority channel reside) and that the high energy regime (*E* > 2.25 eV) is effectively flat. We conclude that the applied field controls these states and excitations through spin-charge interactions.

In order to provide additional information on how these light-generated excitations can be controlled, we carried out a series of magnetic field sweeps of the dichroic response and compared the results to the magnetization of CoFe_2_O_4_ [Fig. 1(d)] which we already know is hysteretic. The latter is expected because spinel ferrites are well-known ferrimagnets, although it is not entirely obvious that the hysteretic nature of the ↓↓↑ to ↑↓↑ transition in CoFe_2_O_4_ will be reflected in the magneto-optical properties. There is little effect near the fundamental indirect gap—mainly because there are so few Co states with which to work. Higher energies are different. Here, a clear hysteresis develops in the MCD response [Figs. 3(a) and 3(b)]. This is important and interesting because the optical tracking of a magnetic hysteresis loop has a number of applications. That the size of the optical hysteresis loop depends upon energy is, however, an unexpected surprise and suggests that the electronic states are spin correlated. Figure 3(c) displays the coercive field as a function of energy. Strikingly, the coercive field determined from optical measurements is overall higher than that extracted from magnetization. It also has a weak dependence on energy. One logical explanation for these observations is that higher energy light accesses more Co states. Overall, the field sweeps of the dichroic response in CoFe_2_O_4_ show that there is a large energy window with promise for ultra-low power devices because of the magnetically switchable optical response.

**FIG. 3.**
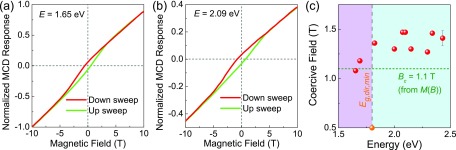
[(a) and (b)] Representative field sweeps of the MCD response for CoFe_2_O_4_ showing the development of the optical hysteresis loop with energy. (c) Magnetic field vs. energy showing changes in the coercive field at 1.6 K. The minority channel direct gap is labeled, and the coercive field obtained from magnetization^12^ is included for comparison.

Motivated by recent work in which iron oxides like BiFeO_3_ are used as active elements of a solar cell,^48^ we decided to take a step toward evaluating CoFe_2_O_4_ for light harvesting applications. Photoconductivity is well suited for this purpose, and it is naturally connected to the series of bandgaps, the spin split electronic structure in spinel ferrites, and the entanglement of the charge and spin. These measurements also provide another opportunity to compare the electronic properties of CoFe_2_O_4_ with those of the Ni analog.^23^

Figure 4 summarizes the photoconductivity of CoFe_2_O_4_. This property derives from the creation of electron-hole pairs with light, *σ*_*PC*_ ∝ *η α*(*E*) *τ*.^49^ Here, *σ*_*PC*_ is the photoconductance, *η* is the probability of creating a carrier, *α*(*E*) is the absorption coefficient, and *τ* is the carrier lifetime. Figure 4(a) displays typical current vs. voltage (*I*–*V*) curves with white light on and off. The open-circuit voltage *V*_*OC*_ is 100 mV at an intensity of ≈50 kW m^−2^. The data in panel (b) were obtained from similar *I*–*V* curves collected at specific illumination energies. Comparison reveals that photoconductivity tracks the absorption spectrum (shown here on a semi-log scale) reasonably well. A closer examination of Fig. 4(b) reveals three regions of particular interest. That centered near 1.0 eV is connected with charge transfer excitations across the fundamental indirect bandgap. There is also a *d*-to-*d* excitation in the vicinity, but a localized excitation will not carry current. *σ*_*PC*_ is largest near 2.0 eV—just above the direct gap in the minority channel. *σ*_*PC*_ continues to rise at energies above the direct gap in the majority channel, with a feature near 3.5 eV that is most likely related to the additional structure in the joint density of states. The non-zero photoconductance below the majority channel direct gap is particularly interesting. It provides evidence that there are indeed important electronic states in the energy window below 2.8 eV arising from the two discrete symmetry environments of the Fe centers. We therefore see that the minority channel states can carry current and that this current can be created with light. A similar situation occurs in NiFe_2_O_4_—although the overall energy scale is higher. The Ni compound also has less structure in *σ*_*PC*_.^40^ Exchange splitting is the origin of spin-dependent excitations in the ferromagnetic insulator Y_3_Fe_5_O_12_ as well.^40,50^

**FIG. 4.**
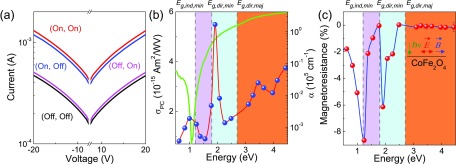
(a) Representative *I*–*V* curves of CoFe_2_O_4_ taken under broadband (white) light at 300 K. Light and magnetic field are indicated to be on or off as (*hν*, *B*). (b) Room temperature photoconductivity of CoFe_2_O_4_ vs. energy, at −20 V, along with the absorption spectrum for comparison. The log scale for absorption emphasizes features below 2 eV. (c) Optically enhanced magnetoresistance of CoFe_2_O_4_ vs. energy at 300 K. The bandgaps in (b) and (c) are 1.2 eV (minority, indirect), 1.8 eV (minority, direct), and 2.7 eV (majority, direct).

The application of a magnetic field provides an opportunity to further explore the photo-excited minority channel carriers. This is because applied field drives a ↓↓↑ to ↑↓↑ transition on the Co sites.^12^ Figure 4(a) displays a typical set of *I*–*V* curves taken under white light. As a reminder, light at this energy excites the Co *O*_*h*_ → Fe *O*_*h*_ charge transfer in the minority channel. The illumination and magnetic field conditions are indicated as (*hν*, *B*). Using *I*–*V* curves like those in Fig. 4(a), we determined field-induced changes in photoconductivity at various energies. Figure 4(c) summarizes these findings by plotting them as magnetoresistances. It is immediately apparent that CoFe_2_O_4_ exhibits strong field effects (−8%) in the range where only minority carriers are active. The strongest effect is near 1.8 eV. This response is well above the standard magnetoresistance (on the order of −1%).^51,52^ We conclude that light and field together are more effective than field alone—at least in the energy window between the minority channel indirect and the majority channel direct gaps. Moreover, magnetoresistance in CoFe_2_O_4_ (−8%) is significantly stronger than that in NiFe_2_O_4_ (−6.5%)—even though the 1.5 T field applied here is not enough to fully saturate the Co moments. Spin-dependent excitations can be manipulated with external electric and/or magnetic fields in Y_3_Fe_5_O_12_ as well.^53^

In summary, we measured the magneto-optical properties of CoFe_2_O_4_ and compared them with prior optical absorption and first principles electronic structure calculations. Analysis of the dichroic response reveals that the full bandgap hierarchy is 1.2 eV (indirect, minority channel), 1.8 eV (direct, minority channel), and 2.7 eV (direct, majority channel). The energy scale is overall lower than that of the Ni analog, and this series of bandgaps has a strong overlap with the solar spectrum. Photoconductivity shows that the minority channel states can carry current, that this current can be created with light, and that it depends upon the magnetic field. Moreover, we show that the applied magnetic field switches the spin state and, by so doing, modifies the electronic properties. Spin-charge coupling, while dramatic in NiFe_2_O_4_, seems to be even more important in the Co compound, probably because the inversion fraction makes a combination of charge transfer excitations more prominent. This work opens the door to new applications of spinel ferrites that employ the magnetic field control of electronic properties.

See supplementary material for the magneto-optical response of the substrate material, MgAl_2_O_4_. We also take a closer look at the derivative relationship between magnetic circular dichroism and optical absorption as well as the lineshape analysis.

## References

[1] J. Philip, A. Punnoose, B. I. Kim, K. M. Reddy, S. Layne, J. O. Holmes, B. Satpati, P. R. LeClair, T. S. Santos, and J. S. Moodera, Nat. Mater. 5, 298 (2006). 10.1038/nmat1613 16547517

[2] J. C. Slonczewski, Phys. Rev. B 82, 0544403 (2010). 10.1103/physrevb.82.054403

[3] K. Uchida, J. Xiao, H. Adachi, J. Ohe, S. Takahashi, J. Ieda, T. Ota, Y. Kajiwara, H. Umezawa, H. Kawai, G. E. W. Bauer, S. Maekawa, and E. Saitoh, Nat. Mater. 9, 894 (2010). 10.1038/nmat2856 20871606

[4] Y. P. Sukhorukov, A. V. Telegin, A. P. Nosov, V. D. Bessonov, and A. A. Buchkevich, JETP Lett. 104, 384 (2016). 10.1134/s0021364016180107

[5] W. G. Fateley, N. T. McDevitt, and F. F. Bentley, Appl. Spectrosc. 25, 155 (1971). 10.1366/000370271779948600

[6] Y. H. Hou, Y. J. Zhao, Z. W. Liu, H. Y. Yu, X. C. Zhong, W. Q. Qiu, D. C. Zeng, and L. S. Wen, J. Phys. D: Appl. Phys. 43, 445003 (2010). 10.1088/0022-3727/43/44/445003

[7] G. A. Sawatzky, J. Appl. Phys. 39, 1204 (1968). 10.1063/1.1656224

[c8] 8.Excursions of *λ* away from 1(0) are in part due to the different ionic radii of Fe^3+^ (0.79 Å) and Co^3+^ (0.75 Å). This structure can be broken up into octants, alternating between tetrahedron and cubes with the 4 O ions in each octant occupying the same orientations.

[9] J. A. Moyer, C. A. F. Vaz, E. Negusse, D. A. Arena, and V. E. Henrich, Phys. Rev. B 83, 035121 (2011). 10.1103/physrevb.83.035121

[10] D. Fritsch and C. Ederer, Phys. Rev. B 86, 014406 (2012). 10.1103/physrevb.86.014406

[11] A. V. Ramos, T. S. Santos, G. X. Miao, M.-J. Guittet, J.-B. Moussy, and J. S. Moodera, Phys. Rev. B 78, 180402 (2008). 10.1103/physrevb.78.180402

[12] J. X. Ma, D. Mazumdar, G. Kim, H. Sato, N. Z. Bao, and A. Gupta, J. Appl. Phys. 108, 063917 (2010). 10.1063/1.3488638

[13] S. Matzen, J.-B. Moussy, P. Wei, C. Gatel, J. C. Cezar, M. A. Arrio, P. Sainctavit, and J. S. Moodera, Appl. Phys. Lett. 104, 182404 (2014). 10.1063/1.4871733

[14] M. Tachiki, Prog. Theor. Phys. 23, 1055 (1960). 10.1143/ptp.23.1055

[15] J. A. Moyer, C. A. F. Vaz, D. P. Kumah, D. A. Arena, and V. E. Henrich, Phys. Rev. B 86, 174404 (2012). 10.1103/physrevb.86.174404

[16] M. D. Sturge, E. M. Gyorgy, R. C. LeCraw, and J. P. Remeika, Phys. Rev. 180, 413 (1969). 10.1103/physrev.180.413

[17] D. Hunter, W. Osborn, K. Wang, N. Kazantseva, J. Hattrick-Simpers, R. Suchoski, R. Takahashi, M. L. Young, A. Mehta, L. A. Bendersky, S. E. Lofland, M. Wuttig, and I. Takeuchi, Nat. Commun. 2, 518 (2011). 10.1038/ncomms1529 22044997

[18] R. V. Chopdekar and Y. Suzuki, Appl. Phys. Lett. 89, 182506 (2006). 10.1063/1.2370881

[19] H. Ryu, P. Murugavel, J. H. Lee, S. C. Chae, T. W. Noh, Y. S. Oh, H. J. Kim, K. H. Kim, J. H. Jang, M. Kim, C. Bae, and J.-G. Park, Appl. Phys. Lett. 89, 102907 (2006). 10.1063/1.2338766

[20] C. Schmitz-Antoniak, D. Schmitz, P. Borisov, F. M. F. de Groot, S. Stienen, A. Warland, B. Krumme, R. Feyerherm, E. Dudzik, W. Kleemann, and H. Wende, Nat. Commun. 4, 3051 (2013). 10.1038/ncomms3051 23797562

[21] H. Zheng, J. Wang, S. E. Lofland, Z. Ma, L. Mohaddes-Ardabili, T. Zhao, L. Salamanca-Riba, S. R. Shinde, S. B. Ogale, F. Bai, D. Viehland, Y. Jia, D. G. Schlom, M. Wuttig, A. Roytburd, and R. Ramesh, Science 303, 661 (2004). 10.1126/science.1094207 14752158

[22] G.-H. Lim, S. Woo, H. Lee, K.-S. Moon, H. Sohn, S.-E. Lee, and B. Lim, ACS Appl. Mater. Interfaces 9, 40628 (2017). 10.1021/acsami.7b12147 29094592

[23] B. S. Holinsworth, D. Mazumdar, H. Sims, Q.-C. Sun, M. K. Yurtisigi, S. K. Sarker, A. Gupta, W. H. Butler, and J. L. Musfeldt, Appl. Phys. Lett. 103, 082406 (2013). 10.1063/1.4818315

[c24] 24.What one seeks from electronic structure calculations is understanding and insight—not a perfect prediction of the bandgap. In our experience, slight differences between predicted and measured gaps are normal; it is the order the different excitations that counts.

[25] N. M. Caffrey, D. Fritsch, T. Archer, S. Sanvito, and C. Ederer, Phys. Rev. B 87, 024419 (2013). 10.1103/physrevb.87.024419

[26] Q. C. Sun, C. S. Birkel, J. Cao, W. Tremel, and J. L. Musfeldt, ACS Nano 6, 4876 (2012). 10.1021/nn301276q 22540958

[27] C. Song, X. J. Liu, K. W. Geng, F. Zeng, and F. Pan, J. Appl. Phys. 101, 103903 (2007). 10.1063/1.2732432

[28] A. Pulido, L. Chen, T. Kaczorowski, D. Holden, M. A. Little, S. Y. Chong, B. J. Slater, D. P. McMahon, B. Bonillo, C. J. Stackhouse, A. Stephenson, C. M. Kane, R. Clowes, T. Hasell, A. I. Cooper, and G. M. Day, Nature 543, 657 (2017). 10.1038/nature21419 28329756 PMC5458805

[29] M. N. Iliev, D. Mazumdar, J. X. Ma, A. Gupta, F. Rigato, and J. Fontcuberta, Phys. Rev. B 83, 014108 (2011). 10.1103/physrevb.83.014108

[30] F. M. Michel, V. Barron, J. Torrent, M. P. Morales, C. J. Serna, J.-F. Boily, Q. Liu, A. Ambrosini, A. C. Cismasu, and G. E. Brown, Proc. Natl. Acad. Sci. U. S. A. 107, 2787 (2010). 10.1073/pnas.0910170107 20133643 PMC2840321

[31] H. Takagi and H. Y. Hwang, Science 327, 1601 (2010). 10.1126/science.1182541 20339063

[32] E. M. Wheeler, B. Lake, A. T. M. N. Islam, M. Reehuis, P. Steffens, T. Guidi, and A. H. Hill, Phys. Rev. B 82, 140406 (2010). 10.1103/physrevb.82.140406

[33] A. Uehara, H. Shinaoka, and Y. Motome, Phys. Rev. B 92, 195150 (2015). 10.1103/physrevb.92.195150

[34] S. Pal and S. Lal, Phys. Rev. B 96, 075139 (2017). 10.1103/physrevb.96.075139

[35] D. Carta, M. F. Casula, A. Falqui, D. Loche, G. Mountjoy, C. Sangregorio, and A. Corrias, J. Phys. Chem. C 113, 8606 (2009). 10.1021/jp901077c 20379573

[36] Z. Yan, D. A. Keller, K. J. Rietwyk, H.-N. Barad, K. Majhi, A. Ginsburg, A. Y. Anderson, and A. Zaban, Energy Technol. 4, 809 (2016). 10.1002/ente.201500402

[c37] 37.Here *λ* quantifies the fraction of divalent cations occupying octahedral rather than (normal) tetrahedral sites.

[38] V. Kocsis, S. Bordács, J. Deisenhofer, K. Ohgushi, Y. Kaneko, Y. Tokura, and I. Kézsmárki (unpublished).

[39] Y. Iwasaki, T. Fukumura, H. Kimura, A. Ohkubo, T. Hasegawa, Y. Hirose, T. Makino, K. Ueno, and M. Kawasaki, Appl. Phys. Express 3, 103001 (2010). 10.1143/apex.3.103001

[40] B. S. Holinsworth, H. Sims, J. G. Cherian, D. Mazumdar, N. C. Harms, B. C. L. Chapman, A. Gupta, S. A. McGill, and J. L. Musfeldt, Phys. Rev. B 96, 094427 (2017). 10.1103/physrevb.96.094427

[41] H. Y. Hwang, Y. Iwasa, M. Kawasaki, B. Keimer, N. Nagaosa, and Y. Tokura, Nat. Mater. 11, 103 (2012). 10.1038/nmat3223 22270825

[42] S. Bader and S. Parkin, Annu. Rev. Condens. Matter Phys. 1, 71 (2010). 10.1146/annurev-conmatphys-070909-104123

[43] M. A. Meeker, B. A. Magill, G. A. Khodaparast, D. Saha, C. J. Stanton, S. McGill, and B. W. Wessels, Phys. Rev. B 92, 125203 (2015). 10.1103/physrevb.92.125203

[44] M. Dobrowolska, K. Tivakornsasithorn, X. Liu, J. K. Furdyna, M. Berciu, K. M. Yu, and W. Walukiewicz, Nat. Mater. 11, 444 (2012). 10.1038/nmat3250 22344325

[c45] 45.A symmetric tensor can be diagonalized by a proper rotation of the coordinate system, and the symmetric components *i* = *j* do not contribute to the magneto-optical effects such as MCD. The antisymmetric (off-diagonal) components, by contrast, do contribute to the MCD response and can be considered to the first order as having a direct dependence upon the magnetization and/or magnetic field.

[c46] 46.The Onsager relation suggests that a symmetric dielectric tensor will have symmetry to off-diagonal components under time reversal upon reversal of the magnetic field, or magnetization, *ε*_*ij*_(*B*) = *ε*_*ji*_(−*B*). Therefore, for each pair of symmetrical *i* ≠ *j* components, they will be proportional to ± components of *B*. Thus, the off-diagonal components correspond to MCD response and should be considered to the first order as having a direct dependence upon the magnetization and/or magnetic field.

[47] G. A. Gehring, M. S. Alshammari, D. S. Score, J. R. Neal, A. Mokhtari, and A. M. Fox, J. Magn. Magn. Mater. 324, 3422 (2012). 10.1016/j.jmmm.2012.02.057

[48] S. R. Basu, L. W. Martin, Y. H. Chu, M. Gajek, R. Ramesh, R. C. Rai, X. Xu, and J. L. Musfeldt, Appl. Phys. Lett. 92, 091905 (2008). 10.1063/1.2887908

[49] P. K. Bandyopadhyay and G. P. Summers, Phys. Rev. B 31, 2422 (1985). 10.1103/physrevb.31.2422 9936054

[50] Y.-N. Xu, Z.-Q. Gu, and W. Y. Ching, J. Appl. Phys. 87, 4867 (2000). 10.1063/1.373185

[51] C. Jin, Q. Zhang, W. B. Mi, E. Y. Jiang, and H. L. Bai, J. Phys. D: Appl. Phys. 43, 385001 (2010). 10.1088/0022-3727/43/38/385001

[52] Z. Quan, W. Liu, X. Li, X. Xu, K. Addison, D. Score, and G. Gehring, Mater. Lett. 65, 2982 (2011). 10.1016/j.matlet.2011.06.027

[53] E. Morosan, D. Natelson, A. H. Nevidomskyy, and Q. Si, Adv. Mater. 24, 4896 (2012). 10.1002/adma.201202018 22893361

